# Endometrial preparation methods prior to frozen embryo transfer: A retrospective cohort study comparing true natural cycle, modified natural cycle and artificial cycle

**DOI:** 10.1007/s00404-021-06371-6

**Published:** 2022-01-30

**Authors:** Lena Mensing, Emilie S. Dahlberg, Bjørn Bay, Anette Gabrielsen, Ulla B. Knudsen

**Affiliations:** 1 Fertility Clinic, Horsens Regional Hospital, Sundvej 30, 8700 Horsens, Denmark; 2 Department of Clinical Medicine, Aarhus University, Palle Juul-Jensens Boulevard 82, Aarhus N, 8200 Aarhus, Denmark; 3 Department of Obstetrics and Gynecology, Aarhus University Hospital, Palle Juul-Jensens Boulevard 99, Aarhus N, 8200 Aarhus, Denmark

**Keywords:** Frozen embryo transfer, Blastocyst transfer, In vitro fertilization, Natural cycle, Modified natural cycle, Programmed cycle

## Abstract

**Purpose:**

The aim of this study was to compare the outcomes of three endometrial preparation methods prior to frozen embryo transfer (FET): Natural cycle (NC), modified natural cycle (mNC), and programmed/artificial cycle (AC) protocols. Primary outcomes investigated were clinical pregnancy rate (CPR) and live birth rate (LBR).

**Methods:**

A retrospective study on 2080 FET cycles including patients ≤ 35 years with a BMI ≤ 30 who underwent FET with a single autologous blastocyst stage embryo at Aarhus University Hospital or Horsens Regional Hospital in the period 2013–2019. Only blastocysts frozen by vitrification were included. No luteal phase support (LPS) was used in natural cycles.

**Results:**

In NC, mNC and AC, CPRs were 34.9%, 40.6% and 32.0%, while LBRs were 32.3%, 36.3% and 26.6%, respectively. There were no significant differences in main outcomes when comparing AC with NC [LBR: OR = 0.9 (0.6; 1.2), *p* = 0.4]. Compared to NC, mNC-FET displayed significantly higher positive hCG, implantation rate, CPR and LBR [LBR: OR = 1.4 (1.0; 1.9), *p* = 0.03]. An analysis with mNC as reference group demonstrated significantly better outcomes in the mNC group compared to AC [LBR: OR 0.6 (0.5; 0.8), *p* =  < 0.01].

**Conclusion:**

The present study overall demonstrated better outcomes including LBR with mNC protocol as compared to NC and AC protocol, while comparison of AC and NC showed both protocols to be equally effective. A programmed cycle may be necessary for women with anovulatory cycles; however, normo-ovulating women may be offered a natural cycle protocol.

**Trial registration number:**

3-3013-3047/1 and 31-1522-44. Date of registration: June 24, 2019 and April 23, 2020.

## Introduction

For several decades, human embryos have been cryopreserved in the practice of assisted reproductive technology (ART) [1]. Most often cryopreservation is performed with embryos that are surplus from previous fresh in vitro fertilization (IVF) cycles in women who undergo fertility treatment. Additionally, frozen embryo transfer (FET) enables perfect timing regarding endometrial receptivity and hormone levels in the patient at the time of transfer. Due to these advantages, elective FET or “freeze-all” cycles are even preferred over fresh cycles in some cases, e.g. when risk of hyperstimulation [2].

While the IVF procedure, identification of the best embryo and the cryopreservation process has gradually improved, the most optimal endometrial preparation for FET cycles still needs to be determined. Synchronization of the endometrium for implantation of the frozen–thawed embryo can be achieved through different preparation protocols, the most common being natural cycle (NC), modified natural cycle (mNC), artificial cycle (AC) FET. The NC protocol does not involve exogenous hormone administration, but ultrasound monitoring of the dominant follicle to detect the exact time of ovulation, and often monitoring of endogenous luteinizing hormone (LH) levels as well. In a mNC, on the other hand, ovulation is triggered by injection of human chorionic gonadotropin (hCG). Application of any natural protocol demands a regular menstrual cycle. Whether exogenous progesterone is given for luteal phase support (LPS) after transfer varies between clinics. In AC-FET, endogenous hormonal secretion is replaced with exogenous estradiol and progesterone administration to prepare the endometrium for implantation and provide early pregnancy support. It varies whether pituitary suppression with Gonadotropin-releasing hormone (GnRH)-agonist prior to estradiol administration is used [3]. The AC protocol, often called programmed cycle, with hormonal replacement therapy is generally recommended for women with anovulatory or irregular cycles but may be an option for normo-ovulating women due to good control in the timing of FET, which is advantageous for both the patient and fertility clinic. However, recent studies have suggested a slight increase in preeclampsia and postpartum hemorrhage in relation to programmed cycles [4–6].

Most previous studies upon the best FET treatment demonstrate live birth rates (LBR) either in favor of (modified) natural cycles [4, 7–10], or no significant difference in LBR between protocols [11–21]. Few have results from true NC as it demands close monitoring. A minority of studies found better LBR in the AC protocol cohort [22, 23].

Therefore, this retrospective cohort study aims to provide additional results in the search for an optimal endometrial preparation method by comparing more than 2000 blastocyst stage single embryo transfers (SET) following a NC protocol versus mNC and AC protocol for endometrial preparation reported on both CPR and LBR.

## Materials and methods

### Patients

The FET cycles included in this retrospective study originate from Aarhus University Hospital and Horsens Regional Hospital, geographically located within 50 km from each other. In 2016, a centralization of fertility treatment in Central Denmark Region was conducted, cumulating patients from the two fertility clinics at Horsens Regional Hospital. Therefore, FET cycles in this study before 2016 originate from Aarhus University Hospital, while the FET cycles from 2016 and onwards originate from the fused patient population at Horsens Regional Hospital. However, the FET treatment was the same at the two hospitals.

A total of 2080 FET cycles from patients ≤ 35 years with BMI ≤ 30 who underwent FET with a single autologous embryo vitrified at blastocyst stage in the period 2013–2019 were included. The women in the included cycles underwent either of the three standard endometrial preparation protocols: NC-FET, mNC-FET, or AC-FET.

### Grading of blastocysts

Transferred blastocysts were graded based on the system developed by Gardner and Schoolcraft [24].

Blastocysts were characterized, on the day of transfer, as high quality if they achieved either of the following scores: 4AA, 4AB, 4BA, 5AA, 5AB, 5BA, 6AA, 6AB, 6BA. Blastocysts with a score of 3AA, 3AB, 3BA, 3BB, 4BB, 5BB or 6BB were good quality blastocysts. Blastocysts with other scores were poor-quality blastocysts.

### Endometrial preparation protocols

The NC-FET and mNC-FET protocols were considered applicable for patients with regular ovulatory cycles of 27–31 days. Per protocol, the treating physician recommended the NC or mNC protocol to women with regular ovulatory cycles, while patients with irregular or anovulatory cycles underwent AC-FET.

#### NC-FET

Patients in the NC-FET group were scanned by transvaginal ultrasound on approximately day 12 of their menstrual cycle to determine the size of the dominant follicle. Once the dominant follicle reached approximately 16 mm, patients were instructed to take LH urine tests every morning to determine the timing of ovulation. The embryo was transferred 6 days after a positive LH test. No LPS was administered.

#### mNC-FET

As the patients in the NC-FET group, patients in the mNC-FET group were scanned by transvaginal ultrasound on approximately day 12 of their menstrual cycle to determine the size of the dominant follicle and thereby the timing of ovulation induction. If the timing of ovulation induction could not be determined on day 12, another transvaginal ultrasound scan was performed 2 days later, or patients received a urinary LH-surge test kit to use in the morning to predict ovulation. Once the dominant follicle reached ≥ 16 mm or on the day of a positive LH test, ovulation was induced by hCG injection (6.500 IE Ovitrelle^®^ or 10.000 IE Pregnyl^®^). The embryo was transferred 6 days after ovulation induction. No LPS was administered.

#### AC-FET

Patients were treated with estradiol (tabl. Estrofem^®^ or Femanest^®^ 2 mg × 3 daily) starting on day 2 of their menstrual cycle. Approximately 15 days after the commencement of estradiol treatment, patients were scanned by transvaginal ultrasound to determine the thickness of the endometrium. If the endometrium did not reach a satisfactory thickness, the dosage of estradiol could be adjusted or supplemented with transdermal Estradiol (Evorel^®^ or Vivelle^®^). Once the thickness of the endometrium reached 8 mm, patients were instructed to commence vaginal progesterone (vag. Crinone^®^ 90 mg × 1–2 daily or Lutinus^®^ 100 mg × 3 daily). The embryo was transferred on day 5 after 4 full days of progesterone supplementation. In the occurrence of pregnancy, detected by s-hCG measurement 11 days after transfer, the hormonal treatment continued until the 8th week of pregnancy.

### Cycle outcome measures

The primary outcomes in this study were CPR and LBR. Clinical pregnancy was defined as the presence of a gestational sac and a fetal heartbeat on an ultrasound scan in the 8th week of pregnancy. Live birth was defined as live born delivery after week 22. The secondary outcomes were rates of positive hCG, implantation and biochemical pregnancy. Positive hCG was defined as s-hCG > 10 IU/l measured 11 days after transfer. The implantation rate was calculated based on the visualization of a gestational sac on transvaginal ultrasound independent of the presence of a fetal heartbeat. A biochemical pregnancy occurred when the patient had a positive hCG test but no evidence of a clinical pregnancy on the ultrasound scan in the 8th week of pregnancy.

### Statistical analysis

All statistical analyses were based on an a priori specified analysis plan and performed using Stata. When comparing demographical variables, Fischer’s exact test was performed on all categorical variables, whilst the continuous variables were tested using either *t* tests (*t* test assuming unequal variances with a two-sided *p* value) or Mann–Whitney tests (two-sample Wilcoxon rank-sum test), as appropriate. Further, we estimated the chance of all outcomes using logistic regression analyses while adjusting for potential confounding variables [maternal age (continuous), smoking (yes/no), alcohol consumption (yes/no), cause of infertility (male/female), blastocyst score (high/good/poor), and cycle number (1/2/3 +)]. A *p* value < 0.05 was considered significant.

## Results

A total of 2080 FET cycles were analyzed. Of these, 378 patients underwent the NC-FET protocol, 650 patients underwent the mNC-FET protocol, and 1052 patients underwent the AC-FET protocol. All FETs were SETs.

### Population characteristics

Population characteristics of the included patients are shown in Table 1.

**Table 1:** Population characteristics according to protocol of frozen embryo transfer

	NC-FET	mNC-FET	AC-FET	*p*
No. FET cycles	378	650	1052	
Age, years (mean ± SD)	30.7 ± 3.0	30.6 ± 3.0	30.0 ± 3.1	0.3
BMI, kg/m^2^ [median (25th/75th percentile)]	22.9 (20.6/26.1)	22.8 (20.7/25.4)	22.7 (20.6/25.9)	0.5
Missing data, *n* (%)	49	10	42	
Smoker, *n* (%)	13 (3.4)	42 (6.5)	61 (5.8)	0.1
Missing data	16	14	31	
Alcohol consumer, *n* (%)	223 (59.0)	293 (45.1)	459 (43.6)	**< 0.01**
Missing data	16	53	69	
Cause of infertility, *n* (%)				
Male factor	231 (71.7)	327 (56.4)	383 (49.6)	**< 0.01**
Female factor	91 (28.3)	253 (43.6)	390 (50.5)	
Missing data	58	70	302	
Method of fertilization, *n* (%)				
IVF	112 (29.6)	337 (51.9)	560 (53.2)	**< 0.01**
ICSI	264 (69.8)	313 (48.1)	491 (46.7)	
Missing data	2	0	1	
Blastocyst score, *n* (%)				
High	208 (55.0)	203 (31.2)	358 (33.8)	**< 0.01**
Good	157 (41.5)	345 (53.1)	529 (50.0)	
Poor	13 (3.5)	102 (15.7)	172 (16.2)	
Cycle number, *n* (%)				
1	206 (54.5)	249 (38.3)	341 (32.4)	**< 0.01**
2	91 (24.1)	195 (30.0)	259 (24.6)	
3+	81 (21.4)	206 (31.7)	452 (43.0)	
Missing data	0	0	0	

Denmark, 2013–2019, *n* = 2080. Bold was used to highlight statistical significance, *p* < 0.05

Regarding lifestyle factors, alcohol consumption levels were significantly higher in the NC-FET group compared to the mNC- and AC-FET groups (59.0%, 45.1% and 43.6%, *p* < 0.01).

Moreover, there were statistically significant differences between the three protocols regarding cause of infertility, method of fertilization, blastocyst quality and cycle number. It is apparent that a male factor accounts for a considerably larger proportion of the cause of infertility amongst patients in the NC-FET group, in comparison with the mNC- and AC-FET groups (*p* < 0.01). This is furthermore reflected in the method of fertilization, where a significantly larger proportion of embryos were fertilized by ICSI in the NC-FET group compared to the mNC- and AC-FET groups (*p* < 0.01).

Significant differences were also seen in the quality of the transferred blastocysts, where a larger proportion of high quality blastocysts and consequently lower proportions of good- and poor-quality blastocysts were seen in the NC-FET group compared to the mNC- and AC-FET groups, which displayed similar qualities of blastocysts (*p* < 0.01). The significant differences in the cycle number among the three endometrial preparation protocols suggest that patients in the NC-FET group were more likely to be in their first cycle, while patients in the mNC- and AC-FET groups were more likely to have attempted one or several cycles before the current one (*p* < 0.01).

No significant differences were found in age, BMI, or smoking status of the patients.

### Outcomes

Table 2 displays the outcome measures following FET in the NC-, mNC- and AC-FET protocols, with NC-FET as the reference group, after adjustment for the abovementioned factors. The positive hCG rate (50.9%), implantation rate (43.3%), clinical pregnancy rate (40.6%) and live birth rate (36.3%) were significantly higher in the mNC-FET group compared to the NC-FET group [OR = 1.4 (1.1; 1.9) *p* = 0.02, OR = 1.4 (1.0; 1.9) *p* = 0.04, OR = 1.5 (1.1; 2.1) *p* = 0.01 and OR = 1.4 (1.0; 1.9) *p* = 0.03, respectively]. However, no significant differences in these measures were seen between the AC- and NC-FET groups [50.0% OR = 1.3 (0.9; 1.7) *p* = 0.1, 36.9% OR = 1.0 (0.7; 1.3) *p* = 0.9, 32.0% OR = 1.0 (0.8; 1.4) *p* = 0.9, and 26.6%, OR = 0.9 (0.6; 1.2) *p* = 0.4, respectively]. The biochemical pregnancy rate was significantly higher in the AC-FET group (18.0%) compared to the NC-FET group (11.9%) [OR = 1.5 (1.0; 2.3) *p* = 0.04]. The biochemical pregnancy rate in the mNC-FET group (10.3%) did not differ significantly from the NC-FET group [OR = 0.9 (0.6; 1.4) *p* = 0.8].

**Table 2:** Adjusted odds ratios following frozen embryo transfer in natural (NC-FET), modified natural (mNC-FET), and artificial cycles (AC-FET)

Treatment outcome	NC-FET	mNC-FET		AC-FET	
	n = 378	n = 650		n = 1052	
	Ref. group				
	*n* (%)	*n* (%)	OR (95% CI) (*p*)	*n* (%)	OR (95% CI) (*p*)
Positive hCG rate	175 (46.3)	331 (50.9)	1.4 (1.1; 1.9) **(0.02)**	526 (50.0)	1.3 (0.9; 1.7) (0.1)
Implantation rate	150 (39.7)	282 (43.4)	1.4 (1.0; 1.9) **(0.04)**	388 (36.9)	1.0 (0.7; 1.3) (0.9)
Clinical pregnancy rate week 8	132 (34.9)	264 (40.6)	1.5 (1.1; 2.1) **(0.01)**	337 (32.0)	1.0 (0.8; 1.4) (0.9)
Biochemical pregnancy rate	43 (11.9)	67 (10.3)	0.9 (0.6; 1.4) (0.8)	189 (18.0)	1.5 (1.0; 2.3) **(0.04)**
Live birth rate	121 (32.3)	231 (36.3)	1.4 (1.0; 1.9) **(0.03)**	276 (26.6)	0.9 (0.6; 1.2) (0.4)

Bold was used to highlight statistical significance, *p* < 0.05

OR adjusted for maternal age, smoking, alcohol consumption, cause of infertility, blastocyst score, and cycle number

Table 3 displays the outcome measures following FET in the NC-, mNC- and AC-FET protocols, with mNC-FET as the reference group after adjustment for the abovementioned factors. The positive hCG rate (46.3%), implantation rate (39.7%), clinical pregnancy rate (34.9%) and live birth rate (32.3%) were significantly lower in the NC-FET group compared to the mNC-FET group [OR = 0.7 (0.5; 0.9) *p* = 0.02, OR = 0.7 (0.5; 1.0) *p* = 0.04, OR = 0.7 (0.5; 0.9) *p* = 0.01 and OR = 0.7 (0.5; 1.0) *p* = 0.03, respectively]. There was no significant difference between the NC- FET group (11.9%) and the mNC-FET group (10.3%) regarding the biochemical pregnancy rate [OR = 1.0 (0.7; 1.6) *p* = 0.84]. When comparing the AC-FET group with the mNC-FET group, significant differences were seen regarding the implantation—[36.9 vs. 43.3%, OR = 0.7 (0.6; 0.9) *p* = 0.01], clinical pregnancy—[32.0 vs. 40.6%, OR = 0.7 (0.5; 0.9) *p* = 0.00], biochemical pregnancy—[18.0 vs. 10.3%, OR = 1.6 (1.2; 2.3) *p* =  < 0.01] and live birth rates [26.6 vs. 36.3%, OR = 0.6 (0.5; 0.8) *p* =  < 0.01], indicating superiority of the mNC-FET protocol. No significant differences were seen between the positive hCG rates [50.0 vs. 50.9%, OR = 0.9 (0.7; 1.1) *p* = 0.28].

**Table 3:** Adjusted odds ratios following frozen embryo transfer in natural (NC-FET), modified natural (mNC-FET), and artificial cycles (AC-FET), with mNC-FET as the reference group

Treatment outcome	mNC-FET	NC-FET		AC-FET	
	n = 650	n = 378		n = 1052	
	Ref. group *n* (%)	*n* (%)	OR (95% CI) (*p*)	*n* (%)	OR (95% CI) (*p*)
Positive hCG rate	331 (50.9)	175 (46.3)	0.7 (0.5; 0.9) **(0.02)**	526 (50.0)	0.9 (0.7; 1.1) (0.3)
Implantation rate	282 (43.4)	150 (39.7)	0.7 (0.5; 1.0) **(0.04)**	388 (36.9)	0.7 (0.6; 0.9) **(0.01)**
Clinical pregnancy rate week 8	264 (40.6)	132 (34.9)	0.7 (0.5; 0.9) **(0.01)**	337 (32.0)	0.7 (0.5; 0.9) **(< 0.01)**
Biochemical pregnancy rate	67 (10.3)	43 (11.9)	1.0 (0.7; 1.6) (0.8)	189 (18.0)	1.6 (1.2; 2.3) **(< 0.01)**
Live birth rate	231 (36.3)	121 (32.3)	0.7 (0.5; 1.0) **(0.03)**	276 (26.6)	0.6 (0.5; 0.8) **(< 0.01)**

Bold was used to highlight statistical significance, *p* < 0.05

OR adjusted for maternal age, smoking, alcohol consumption, cause of infertility, blastocyst score, and cycle number

In the present study, there was a significant difference in cancellation rates (*N* = 347 cycles), which were 10.8%, 20.6%, and 11.1% for NC, mNC and AC, respectively (*p* value < 0.01). The observed difference in the two natural cycle protocols (11.1 vs. 20.6%) seems surprising but may be due to the smaller number of patients in the NC-FET group. The main reason for cancellation was due to the lack of a fair quality embryo after thaw (4.9%, 6.1%, 7.4%, respectively). Logically, insufficient follicular development (4.2%, 7.3%, 0%) and LH-surge/clinical ovulation (1.2%, 5.6%, 0%) appeared to be main reasons in the natural cycle groups but not in AC. Other reasons for cancellation were insufficient endometrial thickness, bleeding, and indication for surgery.

## Discussion

This retrospective cohort study included more than 2000 cycles in patients  ≤ 35 years with BMI ≤ 30, who underwent single FET with autologous vitrified blastocysts following a NC, mNC, or AC protocol. None of the natural cycles used LPS. The results of this study indicate that patients treated by the mNC protocol had higher positive hCG rate, implantation rate, CPR, and LBR compared to patients undergoing FET following a NC, with no significant difference in biochemical pregnancy rate. No statistically significant differences in outcomes were demonstrated between AC-FET and NC-FET except for biochemical pregnancy rate, which was higher in the AC-FET group.

A subsequent analysis with mNC as reference group demonstrated superiority of the mNC protocol in all outcomes compared to AC protocol, except for positive hCG rate, which was not significantly different between the two groups.

Most previous studies on this topic are retrospective cohort studies, and the number of studies including all three protocols, blastocyst stage FET solely and LBR is limited.

In line with the present study, Alur-Gupta et al. demonstrated equivalent CPR and LBR when comparing NC and AC protocol (LBR 45 vs. 46%) [13]. Melnick et al. [7] found that mNC-FET in ovulatory women is associated with increased ongoing pregnancy rates and LBRs compared to AC-FET in anovulatory women (CPR 66.2 vs. 43.8%; LBR 63.1 vs. 37.5%).

In contrast, Lathi et al. [12] demonstrated no difference in LBR comparing mNC vs. AC (27.7 vs. 23.6%). Including ovulatory women only in a small retrospective study, Kim et al. (NC, mNC, AC) [25] likewise found no significant difference between protocols regarding CPR, while LBR was not investigated.

Meanwhile, other studies indicate superiority of the NC protocol, such as a large Australian study by Pakes and colleagues who investigated both CPR and LBR in NC vs. AC-FET (CPR 30.8 vs. 26.1%; LBR 24.4 vs. 18.9%) [26]. Chang et al. [27] compared CPR in NC, mNC and AC-FET with NC-FET as reference group. In contrast to the present study, the CPR was significantly higher in NC compared to AC-FET (41.9 vs. 30.4%), while no significant difference was found between mNC and NC-FET (41.8 vs. 41.9%).

On the other hand, a subanalysis on blastocyst stage FET by Zheng et al. [23] compared AC with NC-FET and showed higher CPR (67.3 vs. 57.0%) and LBR (58.8 vs. 49.7%) in the AC group.

Considering previously published literature on endometrial preparation methods, a certain heterogeneity between studies complicates comparison. Inclusion criteria regarding ovulatory status and patient age vary, as does the quality, stage, and number of transferred embryos. Moreover, protocols variate both in method of cryopreservation, and the use of pituitary suppression or LPS. Only a few randomized controlled trials (RCTs) exist on this topic.

One of them is the large ANTARCTICA-trial by Groenewoud et al. [11], in which the authors found no difference in LBR comparing mNC with AC (11.5 vs. 8.8%). In this study, only 7.6% of the transferred embryos were blastocyst stage embryos. A higher cancellation rate was demonstrated in AC-FET (20.4 vs. 26.7%). Greco et al. [28] included only blastocysts in a somewhat smaller RCT. No significant difference in LBRs was found between the mNC protocol and AC protocol with GnRH-agonist pituitary suppression (45.8 vs. 41.5%). Likewise, Agha-Hosseini and colleagues [18] found mNC and AC to be equally effective in cleavage-stage embryos (LBR 35.3 vs. 31.8%). All RCTs obviously included patients with regular menstrual cycles only.

Last, a very recent systematic review by Mumusoglu et al. [29] on this topic based on data derived firstly from RCTs, meta-analyses, and secondly from large prospective cohort studies suggests a trend towards natural cycle protocols (NC and mNC) being superior to AC.

Several studies have focused on embryo transfer in natural cycles specifically. The close molecular similarities between LH and hCG make it possible to use hCG as an ovulation trigger in mNC. During a natural conception cycle, hCG is produced by the blastocyst and sustains the corpus luteum and thus production of progesterone. Keeping this physiological effect in mind, it is logical to assume that exogenous hCG initially provides inherent additional LPS. On the other hand, precocious exposure to hCG may decrease the endometrial receptivity to embryonic hCG due to receptor-downregulation, as addressed by Evans et al. [2]*.*

However, a recent randomized controlled trial by Mackens et al. [30] compared pregnancy rates of NC-FET vs. mNC-FET. As in the present study, neither of the two natural cycle protocols included LPS, but embryos were not blastocyst stage. The results demonstrated no significant difference in pregnancy rates in natural cycles with or without hCG trigger. This is supported by previous meta-analyses that have compared CPR and LBR in NC vs. mNC [3, 31]. In contrast, a RCT by Fatemi et al. [32] has shown superiority of the NC as compared to mNC in cleavage-stage FET without LPS. Certainly, the use of hCG trigger facilitates the clinical work associated with FET.

Recent evidence indicates that mild ovarian stimulation cycle FET [stimulation with follicle stimulating hormone (FSH), letrozole, or clomiphene citrate] can be performed with comparable results in FET [33, 34], but there are only few data on this. In patients with polycystic ovary syndrome (PCOS), stimulated cycles could be an alternative option to artificial cycles [35, 36].

Strengths of our study include the large number of FET cycles reporting on all three protocols, and on both CPR and LBR. Moreover, inclusion of blastocyst-stage single FETs only, cryopreserved by vitrification, makes this study specific.

To our knowledge, the present study is unique in that respect that LPS was not part of our natural cycle protocols. Exploitation of the endogenous hormone secretion makes a natural cycle protocol appealing for women in whom this approach is possible, namely normo-ovulating women. In addition, our CPR and LBR were not lower compared to studies using LPS, suggesting that the use of exogenous progesterone in natural cycles is unnecessary.

Other strengths are that the universal Danish Health Care System provides income-independent access to medical treatment, hereunder fertility treatment, which minimizes the risk of selection bias. Data was collected prospectively by professional staff members, which is why potential misclassification would not be differential. We adjusted for potential confounders, yet residual confounding of unknown factors will still be a risk.

Several limitations exist within our study. Generally, the retrospective nature of the study can limit our findings. The fertility clinic is not automatically informed about pregnancy outcomes such as LBR, as patients go back to the general obstetric routine care after achieving pregnancy at the fertility clinic. Unfortunately, data on LBR is missing for 4% (*N* = 30 cycles) with confirmed CPR. Although this only comprises 1.4% of the total population, it may still influence the conclusion. Likewise, the missing data on e.g. diagnosis would have been interesting to achieve, however, the proportion of diagnosis would most likely differ between the enclosed patients in the natural and artificial cycles anyway. Furthermore, the subsequent analysis with mNC-FET as reference group (Table 3) contains a risk of type II statistical error. Last, a significant higher cancellation rate in mNC may have influenced our outcomes.

In conclusion, the present study demonstrated higher CPR and LBR in mNC-FET as compared to NC-FET, although statistical significance in LBR was just reached and therefore close to equal. Comparison of NC-FET vs. AC-FET showed no significant difference in CPR and LBR between the two protocols. A subsequent analysis performed to compare mNC with AC protocol demonstrated higher CPRs and LBRs in the mNC-FET group.

A programmed cycle may be necessary for women with anovulatory cycles, as well as for women with severe ovarian insufficiency. However, the slightly increased risk of preeclampsia in programmed cycles suggests that normo-ovulating women should be offered the NC or mNC protocol [5]. Even though the cancellation rate appeared to be significantly higher in mNC as compared to AC, most FETs are completed as scheduled, and unnecessary medication is avoided. For women with irregular cycles (e.g. PCOS), mild ovarian stimulation may be considered as an alternative to artificial cycle protocol. The patient’s preference, clinical feasibility as well as cost-effectiveness are factors that likewise should be acknowledged when it comes to the choice of protocol in FET. Future studies will hopefully further illuminate which protocol is most suitable for the individual patient.

## Data Availability

Requests on data sharing can be made by contacting the corresponding author. Data will be shared after review and approval by the trial scientific board and terms of collaboration will be reached together with a signed data access agreement.
